# Top-Hat HELLISH-VCSOA for optical amplification and wavelength conversion for 0.85 to 1.3μm operation

**DOI:** 10.1186/1556-276X-7-525

**Published:** 2012-09-25

**Authors:** Faten Adel Ismael Chaqmaqchee, Naci Balkan, Jose Maria Ulloa Herrero

**Affiliations:** 1 School of Computer Science and Electronic Engineering, University of Essex, Colchester, CO43SQ, UK; 2 Institute for Systems Based on Optoelectronics and Microtechnology (ISOM), Universidad Politécnica de Madrid, Madrid, 28040, Spain

**Keywords:** HELLISH, VCSOA, GaInNAs, Luminescence, Amplification

## Abstract

The Top-Hat hot electron light emission and lasing in semiconductor heterostructure (HELLISH)-vertical cavity semiconductor optical amplifier (VCSOA) is a modified version of a HELLISH-VCSOA device. It has a shorter p-channel and longer n-channel. The device studied in this work consists of a simple GaAs p-i-n junction, containing 11 Ga_0.35_In_0.65_ N_0.02_As_0.08_/GaAs multiple quantum wells in its intrinsic region; the active region is enclosed between six pairs of GaAs/AlAs top distributed Bragg reflector (DBR) mirrors and 20.5 pairs of AlAs/GaAs bottom DBR mirrors. The operation of the device is based on longitudinal current transport parallel to the layers of the GaAs p-n junction. The device is characterised through *I*-*V*-*L* and by spectral photoluminescence, electroluminescence and electro-photoluminescence measurements. An amplification of about 25 dB is observed at applied voltages of around *V* = 88 V.

## Background

Vertical cavity semiconductor optical amplifiers (VCSOAs) are a topic of increasing interest [1-3] for applications in optical communications. In principle, a VCSOA is a modified vertical cavity surface emitting laser with a reduced top mirror reflectivity and is driven below the lasing threshold. VCSOAs are potentially low-cost alternatives to in-plane SOAs. Unlike the SOAs, they are inherently polarisation insensitive, have high fibre-coupling efficiency, low power consumption, low temperature sensitivity, low noise figure and offer the possibility of fabrication and on-chip testing in two-dimensional arrays [4,5].

Long-wavelength GaInNAs/GaAs quantum well (QW)-based [6] VCSOAs were originally proposed as replacements for GaInAsP/InP QW due to its reduced temperature sensitivity and performance degradation [7,8]. In addition, their growth on GaAs and their integrability with GaAs/Al(Ga)As distributed Bragg reflectors (DBRs) allowed them to be considered as the active region in 1.3-μm vertical cavity devices.

Similar with conventional VCSOAs, the Top-Hat hot electron light emission and lasing in semiconductor heterostructure (THH)-VCSOA has a high bottom AlAs/GaAs DBR reflectivity in excess of 99%. The top AlAs/GaAs DBR has a lower reflectivity at around 60% [9]. The epitaxial structure of the THH-VCSOA is shown in Table 1, consisting of 11 Ga_0.35_In_0.65_ N_0.02_As_0.08_/GaAs QWs that are enclosed between the p- and n-junctions. THH-VCSOAs can be operated as bi-directional wavelength converters, optical amplifiers or modulators [10,11].

**Table 1:** Epitaxial structure of the THH-VCSOA

**Repetition**	**Material**	**Layer thickness (Å)**	**Dopants**	**Concentration/cm**^**3**^
6	GaAs	956	Undoped	-
	AlAs	1,113		
1	GaAs	1,500	C	1 × 10^17^
11	GaAs	100	Undoped	-
	GaInNAs	60		
	GaAs	100		
1	GaAs	1,500	Si	1 × 10^17^
1	AlAs	1,113	n-doped	1 × 10^17^
	GaAs	956		
19	AlAs	1,113	Undoped	-
	GaAs	956		
1	AlAs	1,113		
Semi-insulating GaAs substrate				

In this work, we demonstrate for the first time the operation of the device at 1.3-μm wavelengths at *T* = 300 K with the GaInNAs/GaAs active region. Optical and electrical pumping (photoluminescence (PL), electroluminescence (EL)) were investigated. By combining the two measurements, an electro-photoluminescence (EPL) technique was also performed, from which the light amplification is obtained. The highest gain was achieved when a voltage of *V* = 88 V was applied.

## Methods

### Device operation

**Figure 1:**
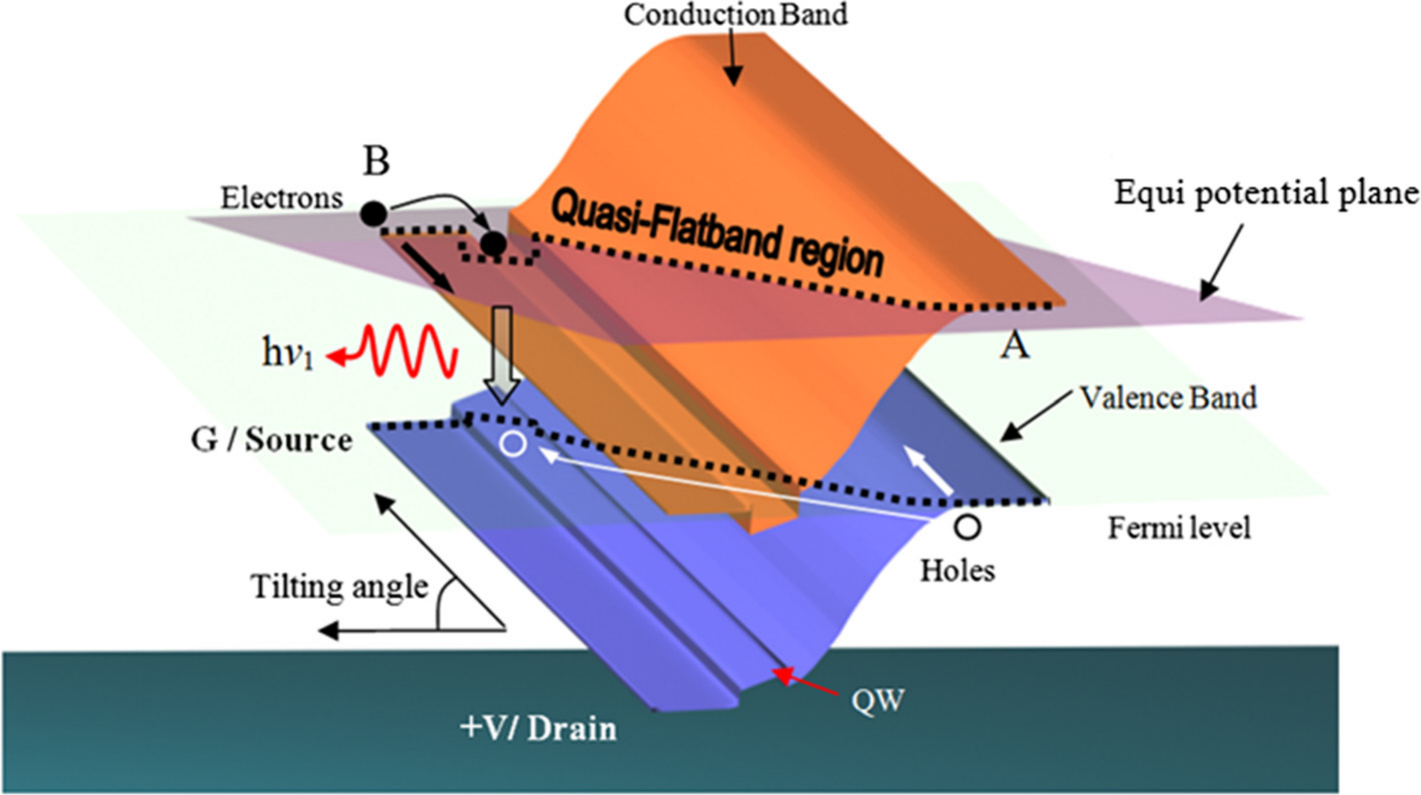
**Three-dimensional model for a standard HELLISH device.** Schematic diagram to illustrate the quasi-flat band region (dotted lines) on the conduction and valence bands. Arrows depict the drifting and diffusion of the holes (white arrow) and electrons (black arrow). The non-filled arrow shows where the radiative recombination takes place, and the emitted photo energy is h*ν*_*1*_.

The operation of the hot electron light emission and lasing in semiconductor heterostructure (HELLISH) device is based on the longitudinal injection of electron and hole pairs in their respective channels. The three-dimensional model for a standard HELLISH device [12] is shown in Figure 1. When the device is biased with low applied voltage, a quasi-flat band is established by tilting the energy bands in the growth path that occupies equal potential states in both channels. In the same way, it is formed diagonally in a forward-biased p-n junction. So, a small number of carriers are able to drift diagonally into the junction plus the longitudinal transport into p-n junction. In this case, the light emission is expected to be weak and depends on the number of holes that arrive in the QWs.

**Figure 2:**
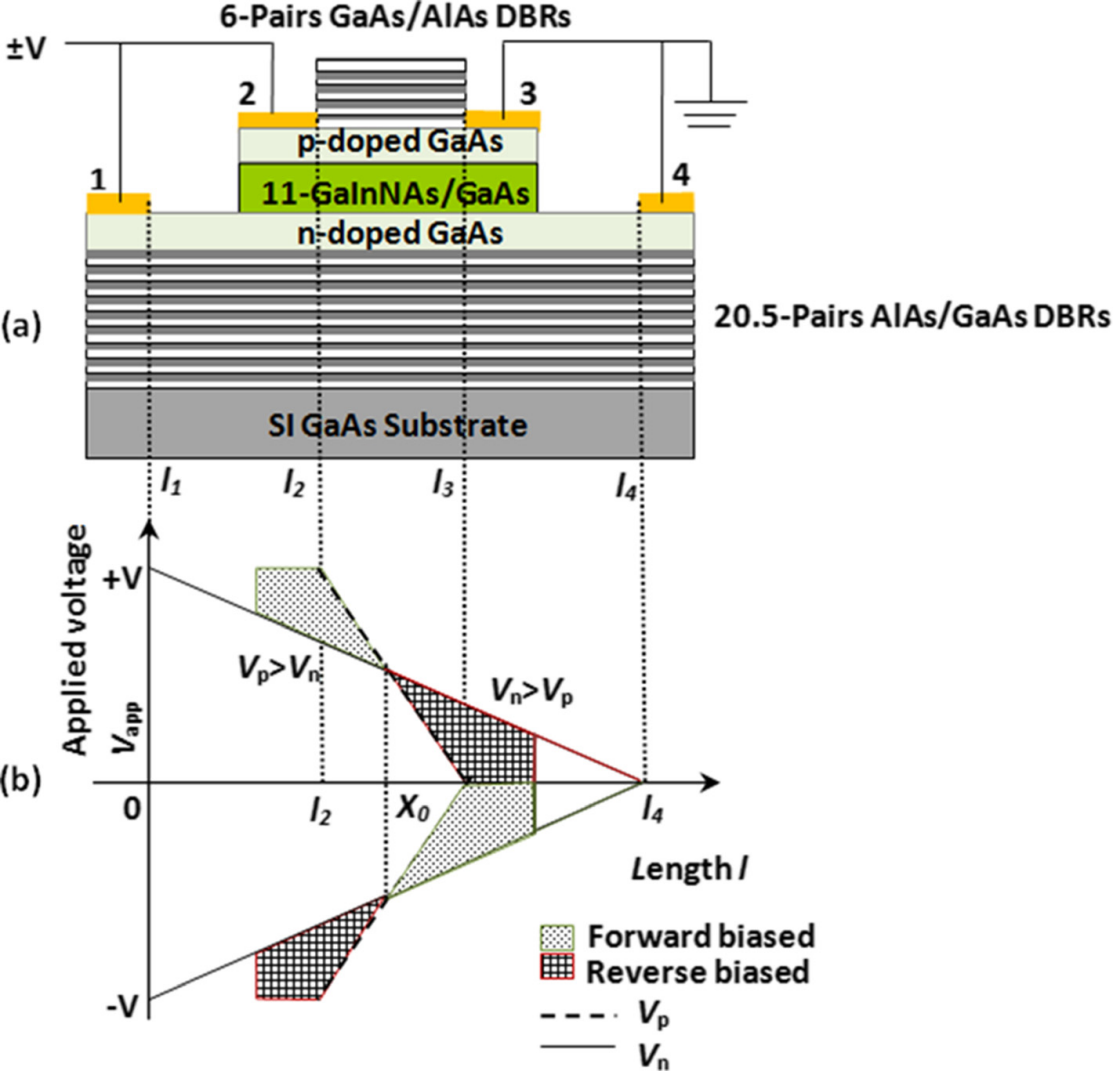
**The modified THH-VCSOA device.** Schematic diagram to illustrate (**a**) the THH-VCSOA structure and its contact configuration and (**b**) the potential distributions of the device along the p-channel (broken line) and n-channel (solid line). In the region of *V*_p_ > *V*_n_, the device is forward-biased, while in the region of *V*_n_ > *V*_p_, the device is reverse-biased.

The THH-VCSOA is an evolution of the standard HELLISH device. It is modified to have a shorter p-channel and a longer n-channel, where the p-channel is located symmetrically with respect to the n-channel, as depicted in Figure 2a. The THH-VCSOA has four contacts enabling the p- and n-channels to be contacted separately but biased with the same voltage.

Under normal process, contacts 1 and 2 on the left side are pulsed with ±V, while contacts 3 and 4 on the right side are grounded (12 ± V34G). By applying a positive voltage (+V), the current flows along the n-channel between contacts 1 and 4. The current flows through the p-channel between contacts 2 and 3. The potential near contact 2 in the p-channel is higher than that in the n-channel (*V*_p_ > *V*_n_), while the situation near contact 3 is the opposite (*V*_n_ > *V*_p_), as shown in Figure 2b. The device, therefore, behaves in two different ways, causing transverse voltage difference across the p-n junction. Firstly, the potential begins to extend from length *l*_*2*_ to point X_0_ and is consequently forward-biased. Thus, this region of the device acts as a light emitter. However, the region from point X_0_ to length *l*_*3*_ is reversed-biased. So, this region of the device acts as a light absorber. When a negative voltage (−V) is applied, the two regions change their functionality. Thus, the device emits light near contact 3 and absorbs light near contact 2. The THH structure has both a forward- and reverse-biased region in the same p-n junction plane, which can be flipped over by changing the polarity of the applied voltage.

### Experimental setup

**Figure 3:**
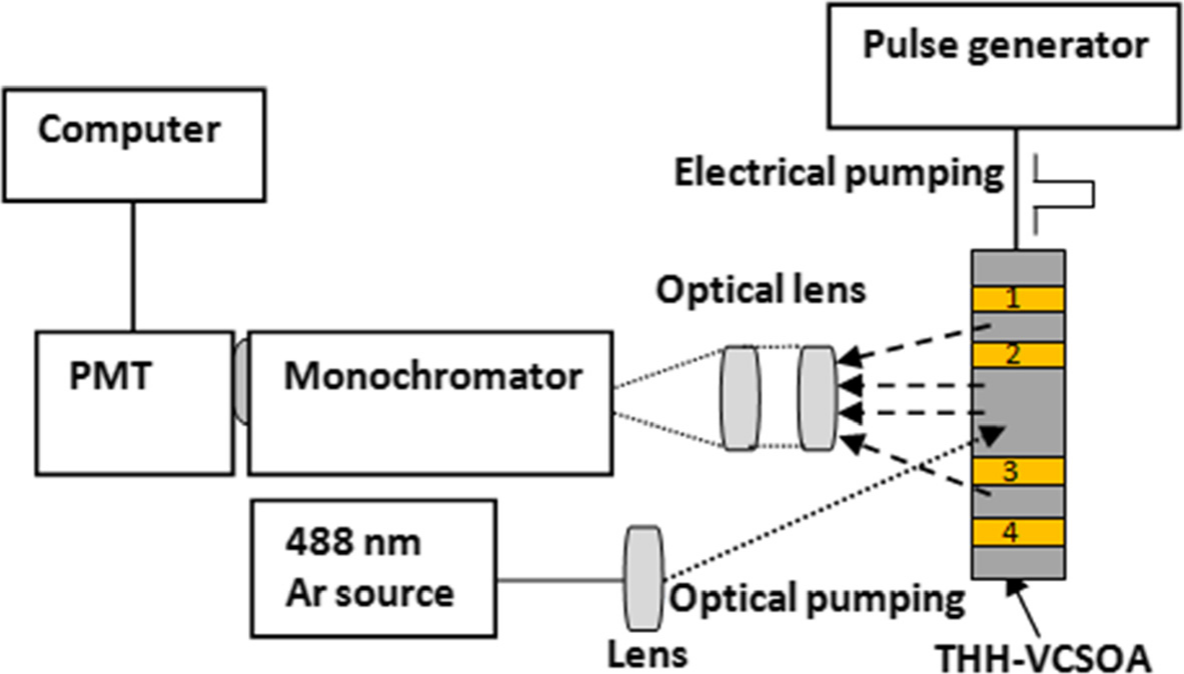
**Experimental setup for THH-VCSOA in reflection mode.** The lengths of the p- and n-channels are 1 and 1.6 mm, respectively.

In order to investigate the functionality of the THH-VCSOA, we used the experimental setup for optical (PL), electrical (EL) and combined optical and electrical (EPL) measurements, as illustrated in Figure 3. In PL and EPL measurements, the optical excitation source is a CW Argon laser operating at a 488-nm wavelength with 17 mW of output power. The laser beam is chopped using a mechanical chopper and directed to the sample surface. The emitted light is dispersed by a Bentham M300 1/3 m monochromator (Bentham, Berkshire, UK) and collected with a cooled InGaAs photomultiplier. The outcoming electrical signal is sent to a Gated Integrator (model 165) and Boxcar Averager Module (model 162, EG&E Princeton Applied Research, Princeton, NJ, USA) and computer system.

## Results and discussion

**Figure 4:**
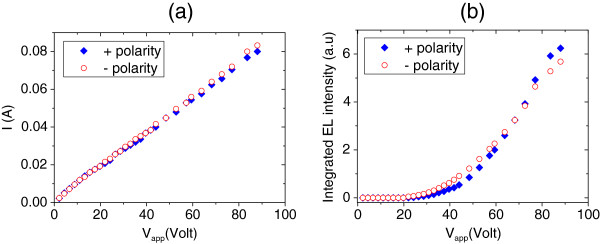
**Measured *I *- *V *and integrated EL intensity versus applied voltages for both polarities at room temperature.** (**a**) Current–voltage and (**b**) integrated EL intensity characteristics for 1- and 1.6-mm-long THH-VCSOA using p- and n-channel configuration (12 ± V34G) under forward and reverse bias at *T* = 14°C.

The THH-VCSOA device investigated in the current work is fabricated with a 1-mm-long p-channel and a 1.6-mm-long n-channel. Figure 4 shows the measured *I*-*V* and the integrated EL intensity versus applied voltages for both polarities at room temperature. The sample is biased with a pulse width of less than 50 μs and repetition rate of slower than 100 Hz to avoid excessive Joule heating. The *I*-*V* characteristic in Figure 4a shows a linear behaviour, implying that both contacts to the n- and p-layers are ohmic. A maximum current of 0.08 A and an applied voltage of 88 V are applied along the layers using both positive and negative polarities. Integrated EL intensity in Figure 4b shows the threshold behaviour with a threshold voltage of around 40 V. It is also clear that the light intensity is independent of the polarity.

**Figure 5:**
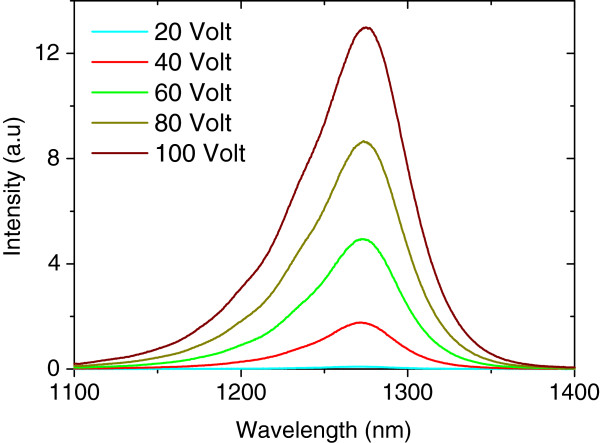
Electroluminescence intensities as a function of applied voltages at a temperature of 14°C.

The emission spectra of EL are plotted as a function of applied voltage in Figure 5. The spectra are taken for negative polarity at 14°C and clearly show the emission peak at around of 1,274 nm.

**Figure 6:**
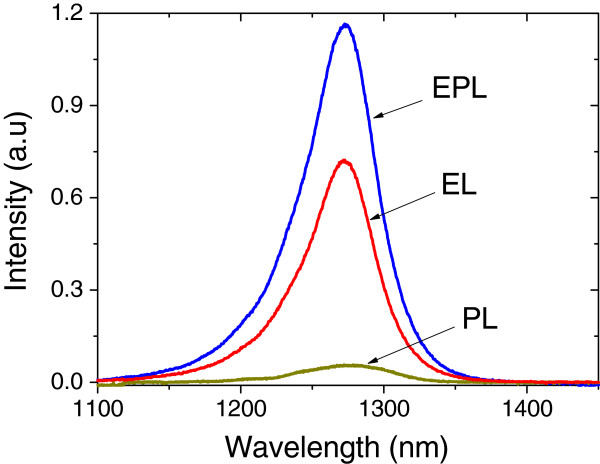
**The EL, PL and EPL intensities measured under applied voltage of 40 V at *T *** **= 16°C.**

The PL, EL and EPL emission spectra of the device have been recorded using optical, electrical and both pumping techniques. The results are shown in Figure 6. The optical pumping is achieved using the 488-nm line of the Ar laser and an input signal power of 17 mW. The device under THH-VCSOA bias configuration is also electrically biased with an applied voltage of 40 V, using pulse width of 50 μs and repetition rate of 9.2 ms. The signal amplification and the emission spectrum from the device are investigated with both optical and electrical pumping. The EL and EPL emission peaks are found to be around 1.27 μm. The spectra have a broad bandwidth of 42 meV due to the fact that light was collected from the whole forward-biased area. The input signal of 488 nm is absorbed by the THH device, causing a modulation of the 1,274 nm-light, thus acting as a wavelength converter. Since the PL has negligible intensity compared to the EL, the difference between the EL and EPL intensities is due to the gain from the device.

**Figure 7:**
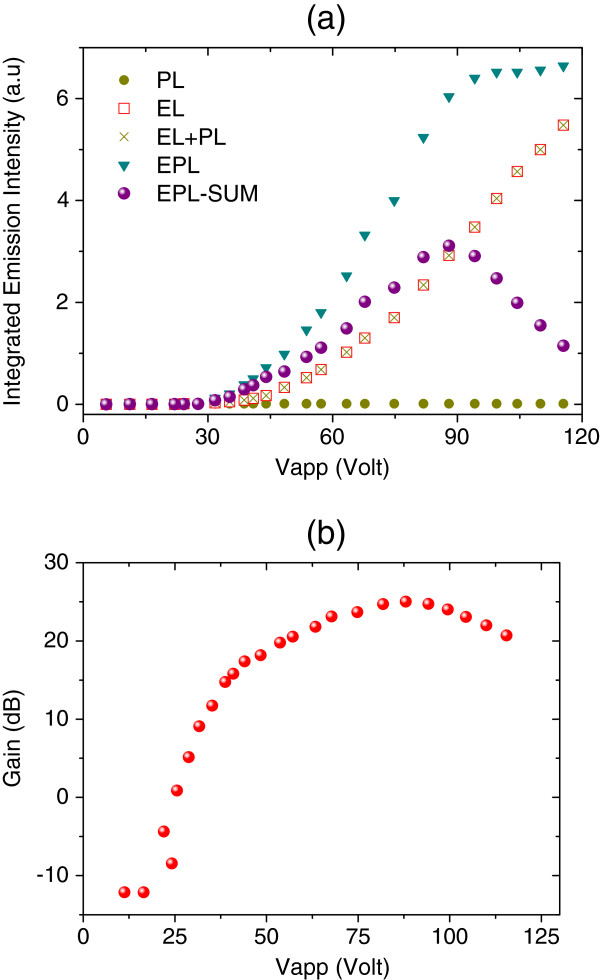
**Integrated PL, EL and EPL intensities and gain as a function of applied voltage.** (**a**) Integrated emission intensities for PL, EL and EPL are measured as a function of applied voltages, together with the calculated EL + PL and EPL-(EL + PL). (**b**) Gain is plotted as a function of applied voltages at *T* = 14°C.

The integrated intensities of PL, EL and EPL, together with the calculated EL + PL and gain, are plotted as a function of applied voltage in Figure 7a. This measurement shows that the intensity of EL is clearly increased when the 488-nm line of the Ar laser is incident onto the sample. It is clear also from the figure that the intensity of EPL-(EL + PL) increases with the applied voltage and then starts to saturate after 88 V due to Joule heating. Figure 7b displays the evolution of gain with the applied voltage, which reaches its maximum at about 25 dB at an applied voltage of 88 V. It should be noted that the wavelength of the excitation laser (488 nm) is very far from the cavity resonance wavelength, thus most of the excitation is lost through the absorption. In addition, the gain here is defined as a ratio of the EPL-(EL + PL) when the device is electrically biased on the PL when the device is not electrically biased.

## Conclusions

The THH-VCSOA device with 1-mm (p-channel) and 1.6-mm (n-channel) contact separation is investigated. The device is characterised using EL, PL and EPL techniques to obtain the amplification. The optical gain at *λ* ~ 1.27 μm is voltage dependent and reaches its maximum of 25 dB at an applied voltage of 88 V. The advantage of using such device is the application of longitudinal electric fields along the active layer, leading to the conclusion that THH-VCSOA works as an optical amplifier and absorber at around the λ = 1.3 μm window of communications.

## Abbreviations

DBR: Distributed Bragg reflector; EL: Electroluminescence; EPL: Electro-photoluminescence; PL: Photoluminescence; QW: Quantum well; VCSOA: Vertical cavity semiconductor optical amplifier; THH: Top-Hat hot electron light emission and lasing in semiconductor heterostructure.

## Competing interests

The authors declare that they have no competing interests.

## Authors' contributions

NB and FAIC designed the structure. FAIC and JMU fabricated the devices. FAIC carried out the experimental work and wrote the article. NB is the inventor of the original device and the overall supervisor of the project. All authors read and approved the final manuscript.

## References

[B1] Kimura T Bjorlin S Piprek J Bowers JE High-temperature characteristics and tunability of long-wavelength vertical-cavity semiconductor optical amplifiers IEEE Photo Tech Lett 2003 15 1501 1503

[B2] Bouche N Corbett B Kuszelewicz R Ray R Vertical-cavity amplifying photonic switch at 1.5 μm IEEE Photo Technol Lett 1996 8 1035

[B3] Bjorlin ES Riou B Abraham P Piprek J Chiu YJ Black KA Keating A Bowers JE Long-wavelength vertical-cavity semiconductor optical amplifiers IEEE J Quant Elect 2001 37 274 10.1109/3.903078

[B4] Coldren CW Larson MC Spruytte SG Harris JS 1200 nm GaAs-based vertical cavity lasers employing GaInNAs multi quantum well active regions Elect Lett 2000 36 951 952 10.1049/el:20000365

[B5] Bjorlin ES Bowers JE Noise figure of vertical-cavity semiconductor optical amplifiers IEEE J Quant Elect 2002 38 61 10.1109/3.973320

[B6] Larson MC Kondow M Kitatani T Tamura K Okai M Photo pumped lasing at 1.25 μm of GaInNAs-GaAs multiple-quantum-well vertical cavity surface emitting laser IEEE Photo Tech Lett 1997 9 1549 1551

[B7] Miyashita N Shimizu Y Okada Y Carrier mobility characteristics in GaInNAs dilute nitride films grown by atomic hydrogen-assisted molecular beam epitaxy J Appl Phys 2007 102 044904 10.1063/1.2770833

[B8] Björlin ES Rious B Keating A Abraham P Chiu Y-J Piprek J Bowers JE 1.3 μm vertical cavity amplifier IEEE Photo Lett 2000 12 951 953

[B9] Chaqmaqchee FAI Mazzucato S Oduncuoglu M Balkan N Sun Y Gunes M Hugues M Hopkinson M GaInNAs-based HELLISH-vertical cavity semiconductor optical amplifier for 1.3-μm operation Nano Res Lett 2011 6 1 7 10.1186/1556-276X-6-104 PMC3211148 21711630

[B10] Wah J-Y Balkan N Boland-thoms A Roberts JS Hot electron light emission and absorption processes in Top Hat structured bi-directional wavelength converter/amplifier Physica E: Low Dimens Syst Nanostruct 2003 1 610 612

[B11] Wah J-Y Boland-Thoms A Balkan N The operation of a novel optically modulated vertical-cavity semiconductor optical amplifier with wavelength converting functionality Phys Stat Sol (c) 2005 2 3100 3103 10.1002/pssc.200460740

[B12] Wah J-Y Balkan N Low field operation of hot electron light emitting devices: quasi-flat-band model IEE Proc Optoelectron 2004 151 483

